# Physiological Roles of Flavodiiron Proteins and Photorespiration in the Liverwort *Marchantia polymorpha*

**DOI:** 10.3389/fpls.2021.668805

**Published:** 2021-08-19

**Authors:** Ginga Shimakawa, Hitomi Hanawa, Shinya Wada, Guy T. Hanke, Yusuke Matsuda, Chikahiro Miyake

**Affiliations:** ^1^Graduate School of Agricultural Science, Kobe University, Kobe, Japan; ^2^Research Center for Solar Energy Chemistry, Osaka University, Suita, Japan; ^3^Department of Biosciences, School of Biological and Environmental Sciences, Kwansei-Gakuin University, Nishinomiya, Japan; ^4^Core Research for Environmental Science and Technology, Japan Science and Technology Agency, Chiyoda, Japan; ^5^School of Biochemistry and Chemistry, Queen Mary University of London, London, United Kingdom

**Keywords:** P700 oxidation, photosynthesis, photorespiration, evolution, oxygen

## Abstract

Against the potential risk in oxygenic photosynthesis, that is, the generation of reactive oxygen species, photosynthetic electron transport needs to be regulated in response to environmental fluctuations. One of the most important regulations is keeping the reaction center chlorophyll (P700) of photosystem I in its oxidized form in excess light conditions. The oxidation of P700 is supported by dissipating excess electrons safely to O_2_, and we previously found that the molecular mechanism of the alternative electron sink is changed from flavodiiron proteins (FLV) to photorespiration in the evolutionary history from cyanobacteria to plants. However, the overall picture of the regulation of photosynthetic electron transport is still not clear in bryophytes, the evolutionary intermediates. Here, we investigated the physiological roles of FLV and photorespiration for P700 oxidation in the liverwort *Marchantia polymorpha* by using the mutants deficient in FLV (*flv1*) at different O_2_ partial pressures. The effective quantum yield of photosystem II significantly decreased at 2kPa O_2_ in *flv1*, indicating that photorespiration functions as the electron sink. Nevertheless, it was clear from the phenotype of *flv1* that FLV was dominant for P700 oxidation in *M. polymorpha*. These data suggested that photorespiration has yet not replaced FLV in functioning for P700 oxidation in the basal land plant probably because of the lower contribution to lumen acidification, compared with FLV, as reflected in the results of electrochromic shift analysis.

## Introduction

To survive natural environmental fluctuations, oxygenic phototrophs have developed a variety of regulatory mechanisms for photosynthetic electron transport. Among these, photoprotection of photosystem I (PSI) is critically important. Without such protection, photo-oxidative damage to PSI derived from reactive oxygen species (ROS) dramatically decreases photosynthetic CO_2_ assimilation and growth rates (Sejima et al., 2014; Zivcak et al., 2015; Shimakawa et al., 2016). Oxidation of the reaction center chlorophyll of PSI, P700, is the universal strategy to suppress ROS production at the electron acceptor side of PSI in excess light conditions, which is regulated by a variety of molecular mechanisms, defined as the “P700 oxidation system” (Shimakawa and Miyake, 2018b). First, the suppression of electron transport at the cytochrome (Cyt) *b*_6_*f* complex contributes to P700 oxidation. The mechanism for this is either through a difference in the proton concentration across thylakoid membrane (ΔpH)-dependent mechanism, by the so-called photosynthetic control (Foyer et al., 1990; Kramer et al., 2003), or through the proposed mechanism dependent on reduced plastoquinone (PQ) pool, termed as RISE (Shaku et al., 2016). Second, an electron sink is required as a prerequisite for P700 oxidation. The electron carrier protein ferredoxin (Fd) transfers the majority of electrons from PSI to the ferredoxin:NADP(H) oxidoreductase (FNR) for reduction of NADP^+^ to NADPH. The dominant electron sink for NADPH is photosynthetic CO_2_ assimilation in the Calvin-Benson-Bassham cycle, which is initiated by the carboxylation reaction of ribulose 1–5 bisphosphate (RuBP) to two molecules of glyceraldehyde 3-phosphate (PGA) by RuBP carboxylase/oxygenase (Rubisco). Also, Rubisco catalyzes the oxygenation reaction of RuBP to generate PGA and 2-phosphoglycolate. The latter one is finally converted to PGA with reduced Fd and ATP in the so-called photorespiration. Photorespiration can therefore function as an electron sink to keep P700 oxidized in C_3_ plants (Furutani et al., 2020b). Flavodiiron proteins (FLV) also function as an alternative electron sink at PSI by transferring electrons from reduced Fd to O_2_ in excess light conditions (Helman et al., 2003; Sétif et al., 2020). Proton gradient regulation 5 (PGR5) and PGR5-like 1 (PGRL1) proteins are required for P700 oxidation but their physiological roles are still elusive (Munekage et al., 2002; DalCorso et al., 2008; Mosebach et al., 2017; Rantala et al., 2020).

The dominant contributors to P700 oxidation have changed over the evolutionary history of the so-called photosynthetic green plastid lineage from cyanobacteria to angiosperms (Shimakawa and Miyake, 2018b; Alboresi et al., 2019). Cyanobacteria, the progenitors of oxygenic photosynthesis, predominantly keep P700 oxidized either by RISE or FLV in excess light conditions, such as fluctuating light and CO_2_ limitation (Allahverdiyeva et al., 2013; Shimakawa et al., 2016). Cyanobacterial genomes encode a homolog of PGR5, but the knockout mutant shows only a minor photosynthetic phenotype (Allahverdiyeva et al., 2013; Sétif et al., 2020), while Dann and Leister reported that cyanobacterial PGR5 functions as plant PGR5 and also as plant PGRL1 (Dann and Leister, 2019). Further, it has been suggested that the cyanobacterial PGR5 homolog is related to redox homeostasis based on a study in which the gene was over-expressed (Margulis et al., 2020). By contrast, the green alga *Chlamydomonas reinhardtii* shows a strong impact of PGR5 on the ΔpH-dependent regulation for P700 oxidation (Johnson et al., 2014; Mosebach et al., 2017; Jokel et al., 2018; Nikkanen et al., 2021). FLV is also important as an electron sink in the green alga (Chaux et al., 2017; Nikkanen et al., 2021), but the genes for FLV are no longer conserved in the genomes of angiosperms (Allahverdiyeva et al., 2015). Genes for photorespiratory metabolism are broadly conserved in photosynthetic organisms (Hagemann et al., 2013), but the *in vivo* activity of photorespiration is negligible in cyanobacteria and many eukaryotic algae (Peltier and Thibault, 1985; Badger et al., 2000). Overall, the clear evidence that photorespiration contributes to P700 oxidation has been only observed in C_3_ plants. Angiosperms strongly rely on photosynthetic control following lumen acidification for P700 oxidation, which is supported by PGR5, PGRL1, and chloroplast NAD(P)H dehydrogenase (Munekage et al., 2002; Suorsa et al., 2012; Yamori et al., 2016; Rantala et al., 2020). One important change from cyanobacteria to angiosperms in the strategy for P700 oxidation is that FLV has been functionally replaced with photorespiration as the dominant electron sink.

Basal land plants, including liverworts, mosses, ferns, and gymnosperms, are assumed to develop the intermediary strategy to keep P700 oxidized between green algae and angiosperms (Shimakawa and Miyake, 2018b; Alboresi et al., 2019). In these land plants, photorespiration keeps photosynthetic linear electron flow at the CO_2_-compensation point (Hanawa et al., 2017), whereas FLV functions for this in cyanobacteria (Shimakawa et al., 2016; Santana-Sanchez et al., 2019). Recently, Storti and co-workers reported that in the moss *Physcomitrium patens* the lack of PGRL1 has little effect on photoprotection except when combined with a mutation in the gene for FLV, which results in photo-oxidative damage of PSI, especially under fluctuating light (Storti et al., 2019; Storti et al., 2020). The PGR5- and PGRL1-dependent regulatory mechanism is therefore assumed to be dispensable for P700 oxidation in the presence of the electron sink mediated by FLV as in the cases that a heterologous expression of FLV complements P700 oxidation in the mutants of *Arabidopsis thaliana* and *Oryza sativa* deficient in PGR5 (Yamamoto et al., 2016; Wada et al., 2018). However, in contrast to C_3_ angiosperms, the contribution of photorespiration to P700 oxidation is poorly understood in these basal land plants.

Here, we sought to investigate the impact of the O_2_-dependent electron sink, i.e., FLV and photorespiration on P700 oxidation in the liverwort *Marchantia polymorpha*, which is positioned between algae and plants in the photosynthetic green plastid lineage (Bowman et al., 2007). One of the habitats of *M. polymorpha* is the marginal area between aquatic and land environments; thus, this plant must anticipate both submergence and drought stress, which presumably exposes *M. polymorpha* to fluctuating O_2_ and CO_2_ availability, because the diffusion coefficient of gasses in water is approximately 10^−4^ times lower than that in the atmosphere (Raven et al., 1985; Badger and Spalding, 2000). As already mentioned above, *M. polymorpha* utilizes both photorespiration and FLV as alternative electron sinks, probably depending on O_2_ availability, because the affinity of Rubisco for O_2_ (*K*_m_ is 300–1,500μm at 25°C) is presumably much lower than that of FLV (*K*_m_ is assumed to be <10μm; Vicente et al., 2002; Orr et al., 2016). A variety of photosynthetic parameters, including chlorophyll fluorescence, P700 absorbance, and the thylakoid membrane potential, was measured at atmospheric (21kPa) and low (2kPa) O_2_ conditions in the *M. polymorpha* wild type (Tak-1) and the mutant deficient in FLV1 (*flv1*) to assess the physiological functions of these molecular mechanisms in regulating photosynthetic electron transport in the basal land plant.

## Materials and Methods

### Culture

A male accession of *M. polymorpha*, Takaragaike (Tak)-1 and the mutant deficient in FLV1 were asexually maintained according to previously described methods (Shimakawa et al., 2017). Tak-1 and each mutant were grown on one-half-strength Gamborg’s B5 agar medium (Gamborg et al., 1968) under a light–dark cycle (14h light, 22°C, 100μmol photons m^−2^ s^−1^, white fluorescent lamp/10h dark, 20°C). For biochemical and physiological measurements, 2-week-old gemmalings were transferred from B5 agar medium into moist vermiculite, and they were grown there for further 2weeks. For the treatment with aminoacetonitrile (AAN), the thalli were floated on tap water with and without 10mm aminoacetonitrile for 3h.

### Oxygen Exchange

Oxygen evolution and uptake of thalli of *M. polymorpha* (2–5cm^2^) were measured in an O_2_ electrode chamber (LD2/3; Hansatech, King’s Lynn, United Kingdom) simultaneously with chlorophyll fluorescence using a Junior-PAM chlorophyll fluorometer (Walz, Effeltrich, Germany). Temperature of the chamber was set to 25°C. Red actinic light was illuminated from the top of the chamber (LH36/2R; Hansatech). Since the O_2_ electrode chamber was a closed system, a CO_2_-saturated condition was simulated by placing a fabric mat wetted with 1M NaHCO_3_ solution below the intact thalli to supply CO_2_ at a concentration of approximately 1kPa.

### Chlorophyll Fluorescence, P700 Absorbance, and ECS

Chlorophyll fluorescence, absorbance of oxidized P700 (P700^+^), and electrochromic shift (ECS) were measured using a Dual-PAM-100 in a 3,010 DUAL gas exchange leaf chamber (Walz). Flowed gasses were saturated with water vapor at 16.0±0.1°C, and the thalli temperature was maintained at 25°C. Ambient air was used for the measurement at atmospheric O_2_ (21kPa) conditions. For the measurement at low O_2_, the standard O_2_ gas at 2kPa in N_2_ was mixed with 1kPa CO_2_ in N_2_ to keep the atmospheric CO_2_ level (approximately 40Pa) in an LI-7000 infrared gas analyzer (Li-COR, Lincoln, United States). Before the measurements, the thalli were adapted to the darkness for 5min.

For chlorophyll fluorescence analysis, a pulse-amplitude modulated red measuring light (620nm, 0.08μmol photons m^−2^ s^−1^) was used (Schreiber et al., 1986; Genty et al., 1989; Baker, 2008). PSII operating efficiency (quantum yield of photochemical energy conversion in PSII), Y(II)=(F_m_' – F')/F_m_'; non-photochemical quenching (NPQ), NPQ=(F_m_ – F_m_')/F_m_'; fraction of “open” PSII centers (with Q_A_ oxidized) on the basis of the lake model for the PSII photosynthetic apparatus, qL=[(F_m_' – F')/(F_m_' – F_o_')]×(F_o_'/F'): F_o_, minimum fluorescence from dark-adapted thalli; F_o_', minimum fluorescence from light-adapted thalli; F_m_, maximum fluorescence from dark-adapted thalli; and F_m_', maximum fluorescence from light-adapted thalli. Red actinic light was supplied using a chip-on-board light emitting diode (LED) array (635nm). Short-saturation pulse light (8,000μmol photons m^−2^ s^−1^, 300ms) was also provided by the LED array for the determinations of F_m_ and F_m_'.

Pulse-amplitude modulated near-infrared measuring lights (830 and 870nm) were applied to measure the transmittance of P700^+^ (Harbinson and Woodward, 1987; Klughammer and Schreiber, 1994). The full oxidation level of P700 was determined by the 300-ms short-saturation flash after 1-s far-red light illumination (730nm), and the full reduction level of P700 was defined in the dark. P700 oxidation was evaluated as the ratio of P700^+^ to the total P700 signal in the light.

ECS was measured with a pulse-amplitude modulated green measuring light (515 and 550nm) using a Dual-PAM-100 fluorometer equipped with a P515-analysis module (Baker et al., 2007; Klughammer et al., 2013). Change in ECS amplitude in the transition from light to dark, reflecting proton motive force, was termed as ECS_total_, which was measured by temporarily turning actinic light off for 600ms during the illumination. The ECS_total_ values were normalized by the magnitude of ECS induced by a 5-μs short-saturation flash (Klughammer et al., 2013). Proton conductance of the chloroplast ATP synthase (*g*_H_^+^) was calculated as the rate constant of the mono-exponential decay of ECS in the transition from light to dark (Sacksteder and Kramer, 2000).

## Results

### Effects of FLV and Photorespiration on Chlorophyll Fluorescence in *M. polymorpha*

In *M. polymorpha*, FLV functions to keep P700 oxidized. Since FLV can mask the effect of photorespiration on P700 oxidation, here, we used the mutant deficient in FLV (*flv1*) that was generated in our previous study in comparison with the wild- type Tak-1 (Shimakawa et al., 2017). There was no difference in net O_2_ evolution rate between Tak-1 and *flv1* in a CO_2_-saturated condition, where photorespiration should not occur, indicating that FLV has no direct impact on photosynthetic CO_2_ assimilation (Shimakawa et al., 2017).

**Figure 1:**
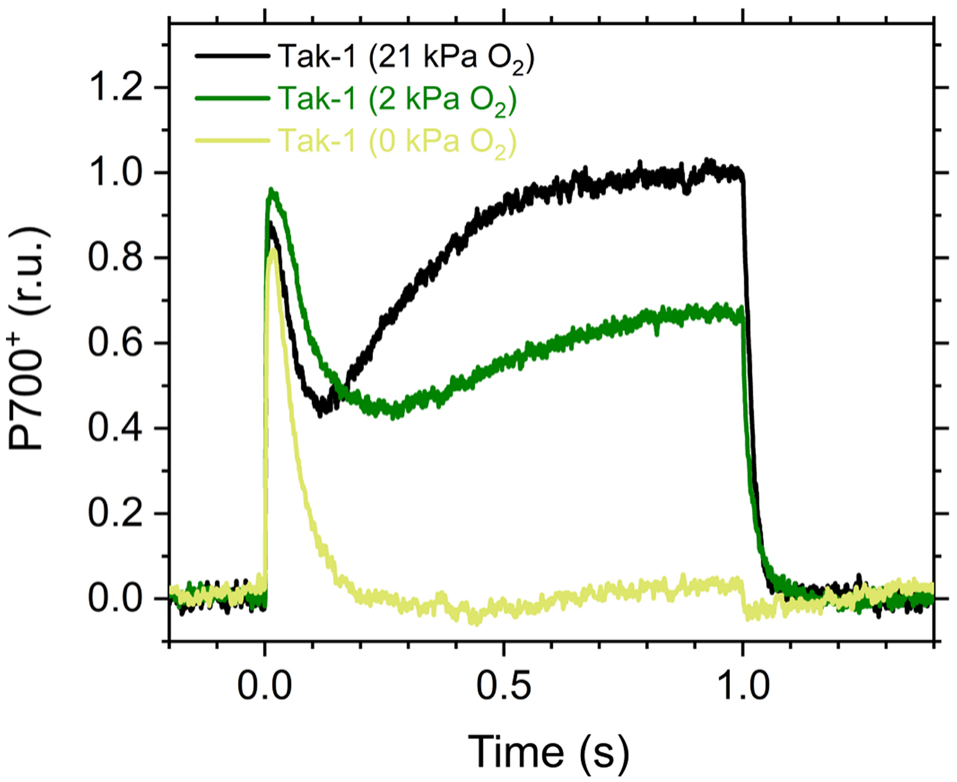
Kinetics of oxidized P700 (P700^+^) in the illumination with a short-pulse light (2,000μmol photons m^−2^ s^−1^, 1s) in the liverwort *Marchantia polymorpha* (Tak-1). Experiments were performed under 21 (black), 2 (green), and 0kPa O_2_ (light green). Relative P700^+^ signals are normalized by the maximum oxidation level of P700 as 1.0. Representative traces of the three independent measurements are shown, respectively.

**Figure 2:**
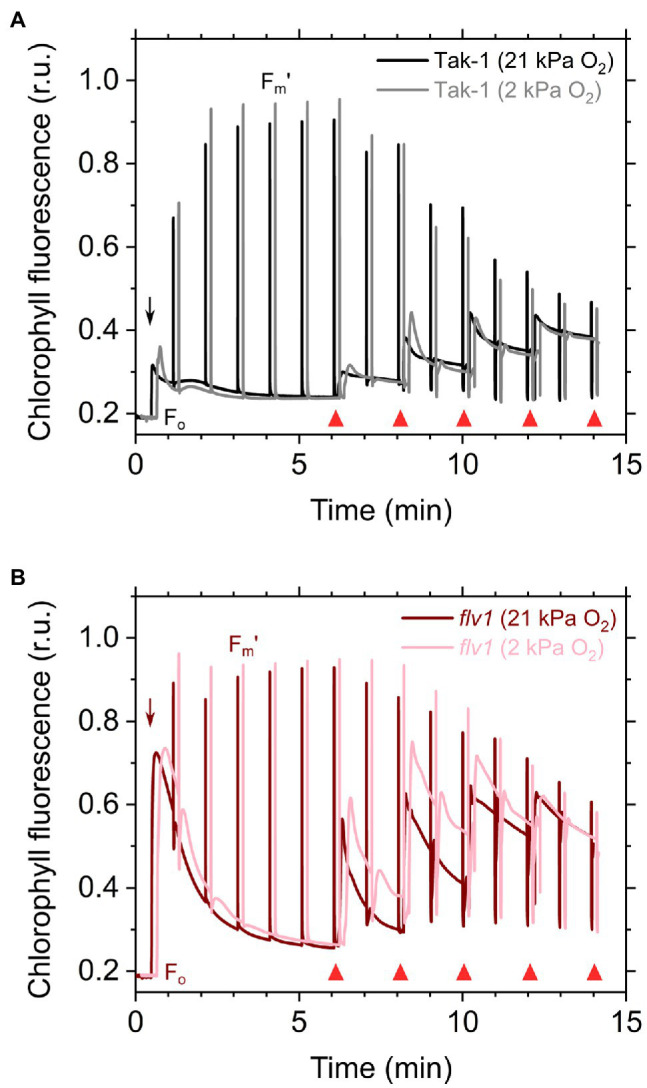
Responses of chlorophyll fluorescence to the light in the liverwort *M. polymorpha* (Tak-1; **A**) and the mutant *flv1* **(B)**. Experiments were performed under 21 (darker lines) and 2kPa O_2_ (lighter lines). Fluorescence measuring light was illuminated to determine the minimum fluorescence from dark-adapted thalli (F_o_). Short-saturation flashes were applied every 1min to determine the maximum fluorescence from light-adapted thalli (F_m_'). Red actinic light was turned on at the time indicated by black arrows, and the light intensities were changed as indicated by red triangles (50, 100, 200, 390, and 760μmol photons m^−2^ s^−1^). Relative chlorophyll fluorescence intensity is normalized by the maximum fluorescence from a dark-adapted thalli (F_m_). Measurements were conducted independently three times (biological replicates), and representative traces are shown.

Here, we analyzed the *in vivo* chlorophyll fluorescence of Tak-1 and *flv1* at atmospheric CO_2_ partial pressure (approximately 40Pa) in atmospheric (21kPa) and low (2kPa) O_2_ conditions. In the atmospheric CO_2_ condition, Rubisco can catalyze the oxygenation of RuBP dependent on the decrease of available CO_2_, driving photorespiration. Because the affinity of Rubisco with O_2_ is low (*K*_m_ is 300–1,500μm at 25°C; Orr et al., 2016), photorespiration should be largely inhibited at 2kPa O_2_ in *M. polymorpha*. Compared with Rubisco, FLV is assumed to show a higher affinity with O_2_ (*K*_m_ is presumed to be a few μm at 25°C; Vicente et al., 2002; Romão et al., 2016). However, we note that the FLV-mediated alternative electron transport was partially inhibited at 2kPa O_2_ in *M. polymorpha* (Figure 1). In Figure 2, the representative raw traces of chlorophyll fluorescence are shown. After the chlorophyll fluorescence intensity reached a stationary level, the actinic light intensity was changed every 2min during the measurements. Compared with Tak-1, *flv1* showed the higher fluorescence intensities especially in the induction phase of photosynthesis and at high light intensities (Figure 2). That is, the deficit of FLV accumulated electrons at the acceptor side of PSII, especially at 2kPa O_2_.

**Figure 3:**
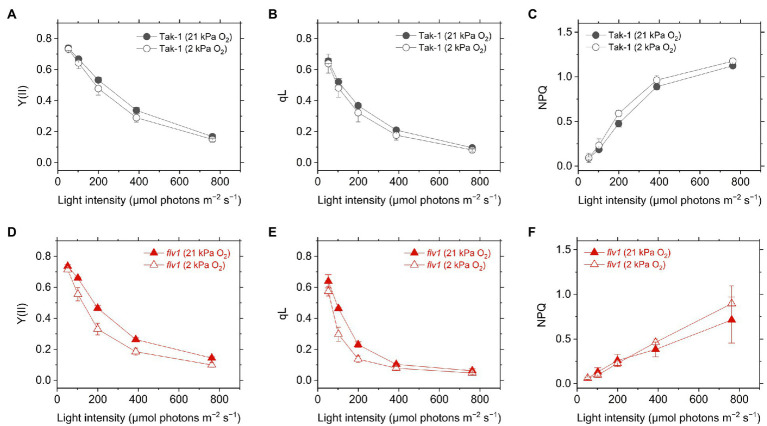
Effective quantum yield of PSII, Y(II) **(A,D)**, inferred oxidation level of plastoquinone pool (qL) **(B,E)**, and non-photochemical quenching (NPQ) of PSII (NPQ; **C,F**) in the liverwort *M. polymorpha* (Tak-1; **A–C**) and the mutant *flv1* **(D–F)**. Experiments were performed under 21 (closed symbols) and 2kPa O_2_ (open symbols). Data are shown as the mean with the standard deviation (*n*=3, biological replicates).

Y(II) calculated from the chlorophyll fluorescence was lower in *flv1*, indicating that FLV functions as the electron sink in *M. polymorpha* (Shimakawa et al., 2017). Here, we note that even in *flv1* Y(II) decreased at 2kPa O_2_ (Figures 3A,B), which suggested that photorespiration functions as the electron sink in *M. polymorpha* (Hanawa et al., 2017). The redox state of the acceptor side of PSII was also evaluated using the chlorophyll fluorescence parameter qL which infers the oxidation level of the PQ pool. Similar trends to Y(II) were observed in Tak-1 and *flv1* in both atmospheric and low O_2_ conditions (Figures 3C,D).

Non-photochemical quenching is the chlorophyll fluorescence parameter showing the amplitude of heat dissipation of light energy at PSII and is assumed to have a correlation with ΔpH in plant leaves (Kanazawa and Kramer, 2002). In both Tak-1 and *flv1*, NPQ slightly increased at 2kPa O_2_, compared with 21kPa O_2_ (Figures 3E,F). Regardless of the O_2_ partial pressure, *flv1* clearly showed lower NPQ than Tak-1.

### Effects of FLV and Photorespiration on P700 Oxidation in *M. polymorpha*

**Figure 4:**
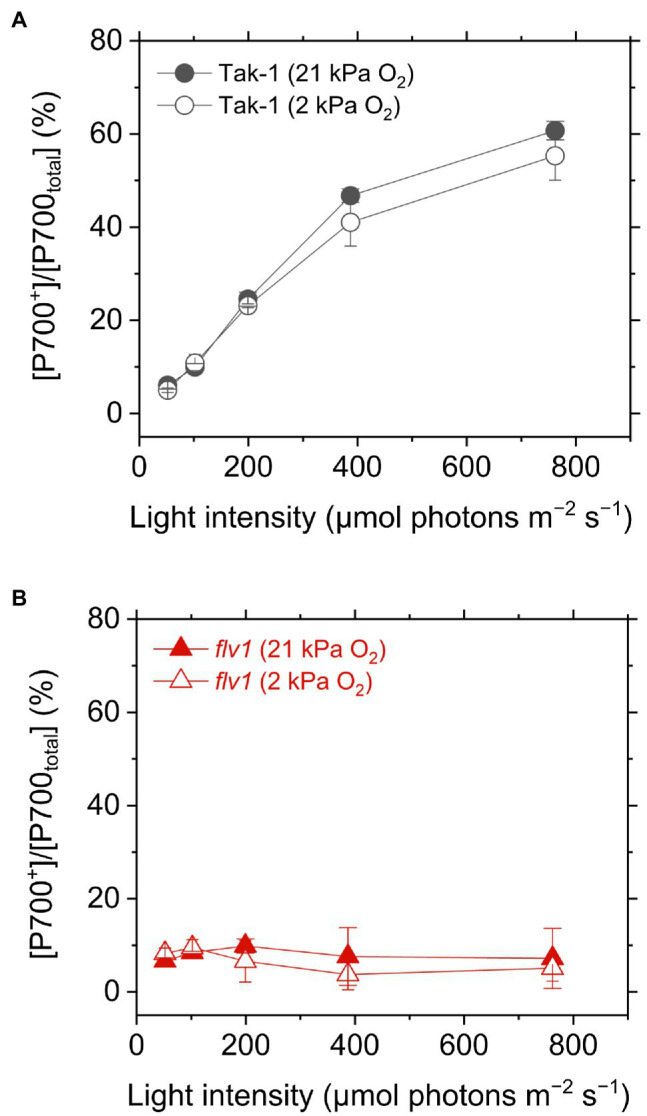
Oxidation of P700 in the liverwort *M. polymorpha* (Tak-1; **A**) and the mutant *flv1* **(B)**. Experiments were performed under 21 (closed symbols) and 2kPa O_2_ (open symbols). Data are shown as the mean with the standard deviation (*n*=3, biological replicates).

**Figure 5:**
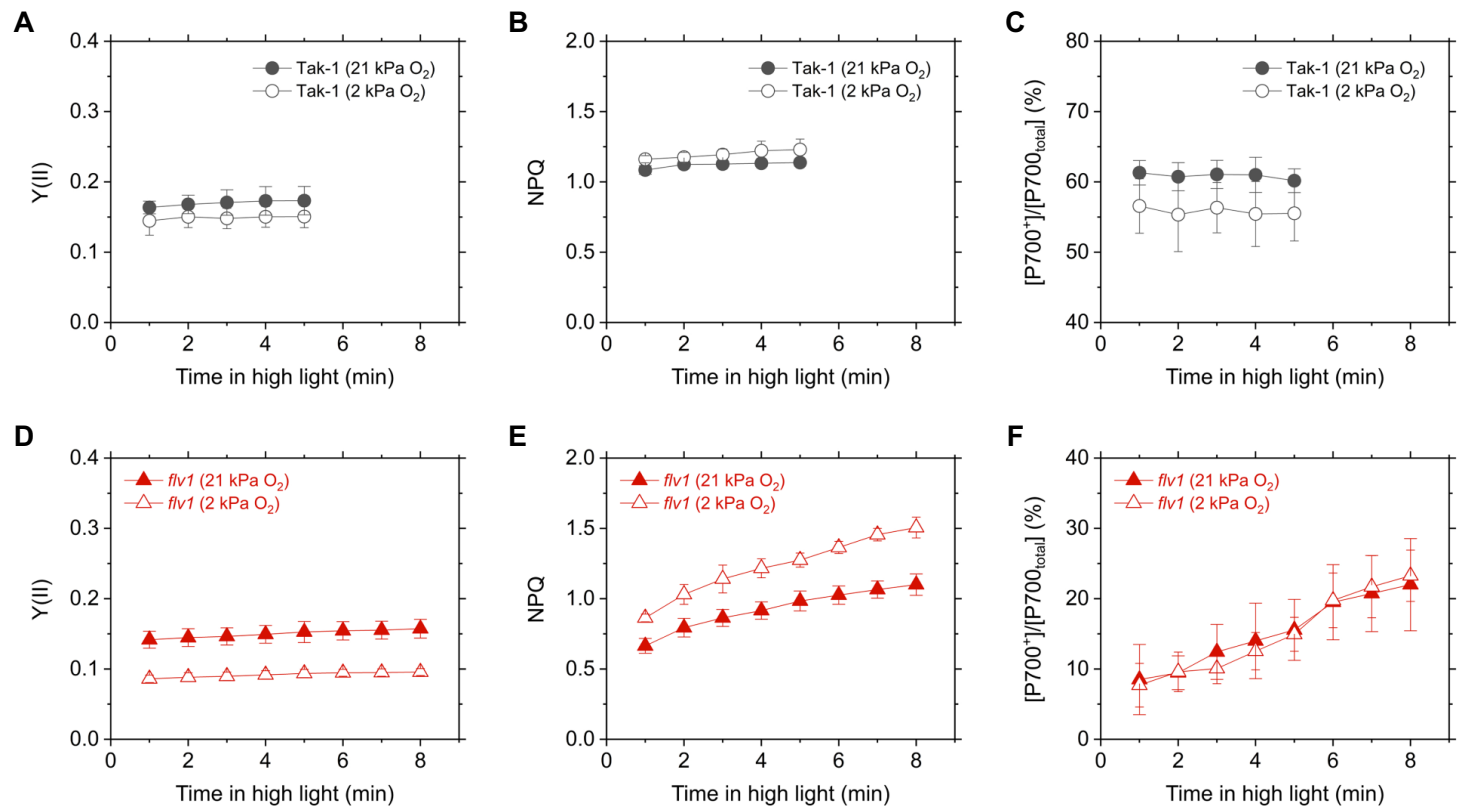
Time-dependent changes of effective quantum yield of PSII, Y(II) **(A,D)**, NPQ **(B,E)**, and P700 oxidation **(C,F)** in high light (760μmol photons m^−2^ s^−1^) in the liverwort *M. polymorpha* (Tak-1; **A–C**) and the mutant *flv1* **(D–F)**. Experiments were performed under 21 (closed symbols) and 2kPa O_2_ (open symbols). Data are shown as the mean with the standard deviation (*n*=3, biological replicates).

We evaluated the redox state of P700 from *in vivo* near- infrared absorbance in Tak-1 and *flv1*. Whereas Tak-1 kept P700 more oxidized at higher light intensities, *flv1* showed little oxidation of P700 at various light intensities regardless of the O_2_ partial pressure (Figure 4). This shows that, in the absence of FLV, the rate determination step in electron transport at PSI is at the acceptor side. However, we note that the prolong illumination at high light intensities promoted P700 oxidation in *flv1* at both 21 and 2kPa O_2_, whereas Tak-1 kept almost the constant oxidized level (Figure 5). That is, *flv1* can partially keep P700 oxidize after a certain time in high light conditions even where photorespiration is suppressed (Shimakawa et al., 2017). In *flv1*, NPQ also increased during the prolong exposure to high light (Figure 5).

### Effects of FLV and Photorespiration on Thylakoid Membrane Potential in *M. polymorpha*

**Figure 6:**
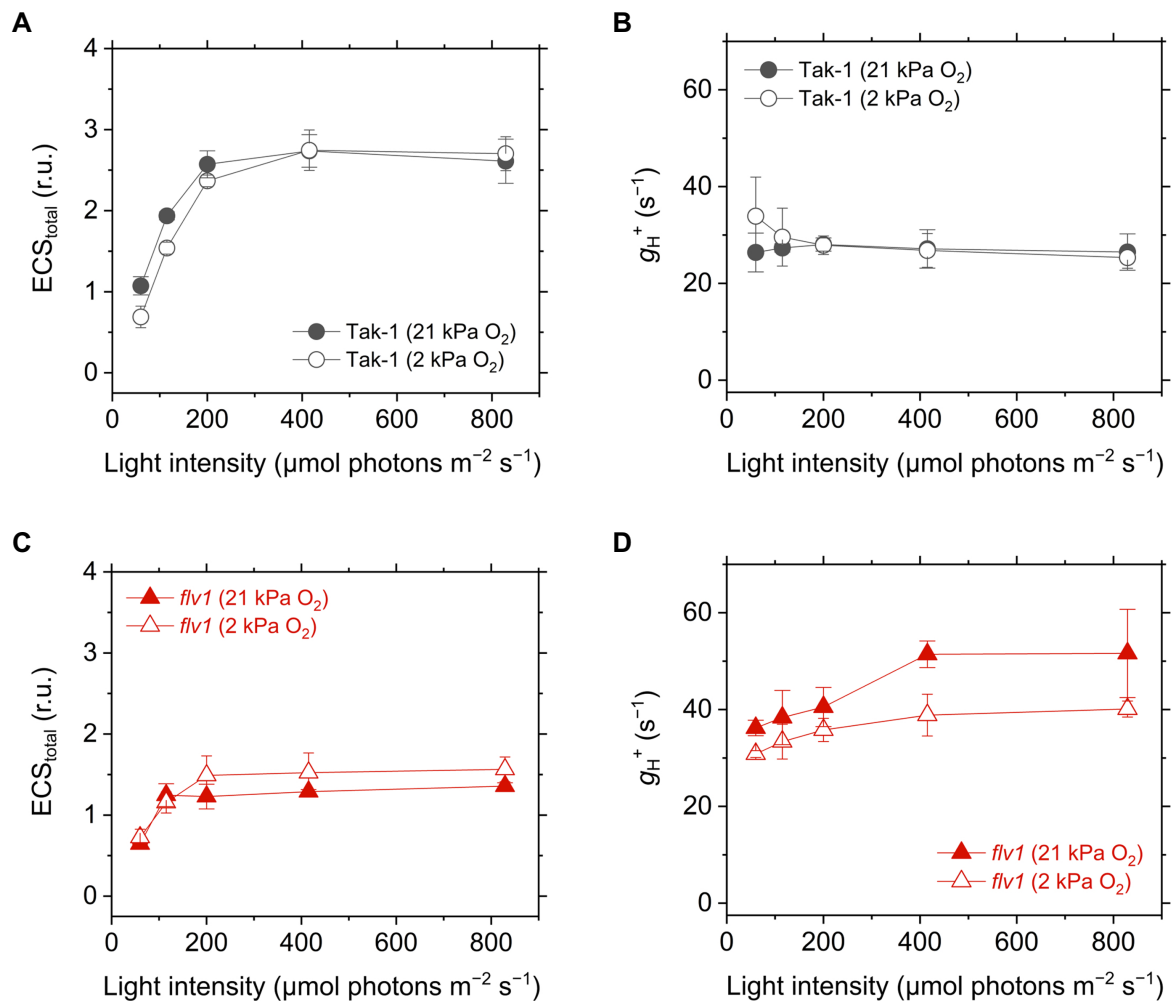
Electrochromic shift (ECS) signal amplitude showing thylakoid membrane potential, termed as ECS_total_ **(A,C)**, and proton conductance of the chloroplast ATP synthase, termed as *g*_H_^+^ **(B,D)**, in the liverwort *M. polymorpha* (Tak-1; **A,B**) and the mutant *flv1* **(B,D)**. The relative ECS values were normalized by the ECS amplitude induced by 5-μs short-saturation flash. Experiments were performed under 21 (closed symbols) and 2kPa O_2_ (open symbols). Data are shown as the mean with the standard deviation (*n*=3, biological replicates).

We analyzed the ECS in Tak-1 and *flv1*. The ECS signal is considered to be an intrinsic optical voltmeter that rapidly responds to changes in the electrical potential across the thylakoid membrane (Witt, 1979). The thylakoid membrane potential during photosynthesis is defined as the total rapid (<1s) change in the ECS signal upon rapidly switching off AL from the steady state, which includes two components: transmembrane differences in ∆pH and in the electric field (∆*Ψ*; Cruz et al., 2001; Johnson and Ruban, 2014). We note that the ECS amplitude depends on the properties of the leaves, not only the density of chloroplasts, but also the content of light-harvesting complexes that house the pigments in which the shift occurs. Therefore, the ECS amplitude was normalized by the magnitude of ECS induced by a 5- μs short- saturation flash, and it was finally termed as ECS_total_ (Klughammer et al., 2013). The *flv1* mutant showed a lower ECS_total_ during steady-state photosynthesis (Figure 6), which is in agreement with the lower Y(II) and NPQ (Figure 3). Proton conductance of the chloroplast ATP synthase, termed as *g*_H_^+^, was also calculated from ECS decay during the transition from light to dark, implying that *flv1* had greater H^+^ leakage from the thylakoid lumen to the stroma than Tak-1 especially at 21kPa O_2_ (Figure 6). One thing that should be noted here is that NPQ is also different between Tak-1 and *flv1* (Figure 3), which may lead to the difference in *g*_H_^+^, independent of H^+^ leakage, since the ECS signal is influenced by the relaxation of NPQ (Johnson and Ruban, 2014).

### Effects of FLV and Photorespiration on the Acceptor Side of PSI in *M. polymorpha*

**Figure 7:**
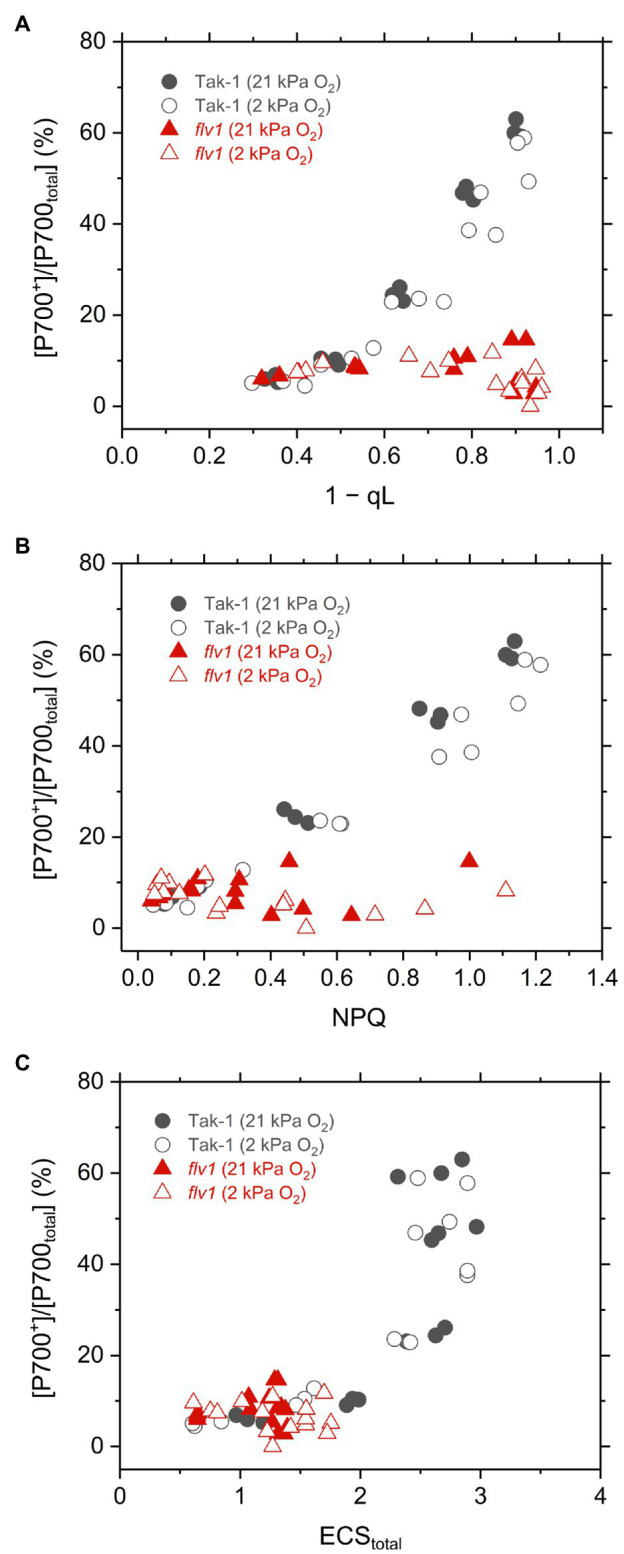
Relationships of P700 oxidation with inferred reduction level of plastoquinone pool (1−qL; **A**), NPQ **(B)**, and thylakoid membrane potential reflected in ECS_total_ **(C)** in the liverwort *M. polymorpha* (Tak-1; black circles) and the mutant *flv1* (red triangles). Experiments were performed under 21 (closed symbols) and 2kPa O_2_ (open symbols). Data are derived from three independent measurements in Figures 3, 4, and 6.

Finally, we plotted the P700 oxidation ratio to 1 – qL, NPQ, and ECS_total_ in Tak-1 and *flv1* to assess the mechanism that FLV contributes to P700 oxidation more than photorespiration (Figure 7). Classically, the impairment of P700 oxidation has been evaluated based on the increase of the acceptor-side limitation of PSI, often termed as Y(NA) (Klughammer and Schreiber, 1994). However, it seems to be difficult to precisely determine Y(NA) and effective quantum yield of PSI, Y(I), using a near-infrared spectrophotometer because of the fast electron transport between plastocyanin and P700 (see “Discussion”). In this study, we evaluated the impairment of P700 oxidation from its relationship with the inferred reduction state of the PQ pool, 1 – qL (Shimakawa and Miyake, 2018a). Since the electron transport is limited in Cyt *b*_6_*f* complex when P700 is kept oxidized, it is theoretically usual that P700 oxidation has a linear relationship with inferred PQ reduction (1 – qL), which was actually recognized in plant leaves and is a useful indicator to evaluate the acceptor side limitation of PSI (Shimakawa and Miyake, 2018a). In *flv1*, the inability to keep P700 oxidized was accompanied with the losses of NPQ and ECS_total_, indicating that the ΔpH-dependent suppression of electron transport in Cyt *b*_6_*f* complex did not function in addition to the loss of the O_2_-dependent electron sink.

## Discussion

In this study, we investigated the roles of FLV and photorespiration in regulating photosynthetic electron transport in the basal land plant *M. polymorpha* using the mutants deficient in FLV1 and PGR5. As shown in the preceding studies in *M. polymorpha* and *P. patens* (Gerotto et al., 2016; Shimakawa et al., 2017; Storti et al., 2019; Storti et al., 2020), FLV is indispensable for keeping P700 oxidized in high light conditions unless the light exposure is prolonged for an acclimation (Figure 5; Shimakawa et al., 2017). Meanwhile, Y(II) and qL decreased at 2kPa O_2_ even in *flv1*, indicating that there is another O_2_-dependent electron sink that should be photorespiration (Hanawa et al., 2017). Although we could not evaluate the pure contribution of photorespiration to P700 oxidation because FLV was also partially inhibited at 2kPa O_2_ (Figure 1), the phenotype of *flv1* obviously showed that FLV is dominant for keeping P700 oxidized in *M. polymorpha* (Figure 4).

Oxygenic phototrophs possess several transport pathways of electrons generated in photosystems to O_2_. First, photorespiratory C_2_ cycle needs one reduced Fd to metabolize one 2-phosphoglycolate generated by the oxygenation reaction of Rubisco and releases each half of molecule of CO_2_ and PGA that are utilized in the Calvin-Benson cycle (Bauwe et al., 2010). Second, FLV donates electrons from reduced Fd to O_2_ (Alboresi et al., 2019). Third, electrons at the acceptor side of PSI can reduce O_2_ also through the Mehler reaction (Miyake, 2010). Fourth, plastid terminal oxidase donates electrons from plastoquinol to O_2_ (Nawrocki et al., 2015). Finally, the mitochondrial respiration possibly functions as an electron sink for photosynthetic electron transport in chloroplasts (Selinski and Scheibe, 2019) although it remains to be assessed in bryophytes. Based on the *in vivo* studies using quantitative gas exchange analyses, photorespiration and FLV have the electron sink capacities enough to replace the Calvin-Benson cycle in various oxygenic phototrophs, including cyanobacteria, green algae, and C_3_ plants at least non-stress conditions (Helman et al., 2005; Driever and Baker, 2011; Allahverdiyeva et al., 2013; Chaux et al., 2017; Burlacot et al., 2018; Santana-Sanchez et al., 2019). Also in bryophytes, Aro and co-workers have found that a large part (*ca.* 70%) of electron flux to O_2_ is attributed to photorespiration (Aro et al., 1984), and the other contributor should be FLV based on the recent knowledges (Gerotto et al., 2016; Shimakawa et al., 2017). Further, the photorespiratory activity has been characterized also by analyzing post-illumination O_2_ uptake at a CO_2_-compensation point in *M. polymorpha* (Hanawa et al., 2017). Therefore, here, we concluded that the decrease of Y(II) at 2kPa O_2_ in *flv1* was attributed to the inhibition of photorespiration. We also tried to inhibit photorespiration using a specific inhibitor aminoacetonitrile (Cho et al., 1985). However, the treatment of the *M. polymorpha* thalli with AAN decreased Y(II) even in a high CO_2_ condition (Supplementary Figure S1) probably due to the accumulation of photorespiratory intermediates toxic to the Calvin-Benson cycle enzymes (Dellero et al., 2016). Therefore, we sought to suppress photorespiration by changing O_2_ partial pressure from 21 to 2kPa in the present study.

Different from C_3_ plants, photorespiration is not dominant for P700 oxidation although it functions as the electron sink in *M. polymorpha*. The extent of the decrease of Y(II) in *flv1* at 2kPa O_2_ was similar to that by the lack of FLV (Figure 3). Rather, photorespiration has the larger electron sink capacity than FLV under CO_2_ limitation (Aro et al., 1984; Hanawa et al., 2017). Nevertheless, it has been clearly shown that FLV is essential for P700 oxidation in *M. polymorpha* (Figure 4). One of the most plausible reason is that FLV does not consume ATP, different from photorespiration, and should contribute to producing ΔpH more than photorespiration, which induces the suppression of electron transport in Cyt *b*_6_*f* complex to support P700 oxidation in addition to the function as the electron sink, as reflected in the higher NPQ and ECS_total_ in Tak-1 at both 21 and 2kPa O_2_ (Figures 3, 6). Meanwhile, P700 oxidation did not depend on the thylakoid membrane potential where ECS_total_ was almost saturated (Figure 7C). This can be interpreted based on the modulation of the balance between ΔpH and ∆*Ψ* (Strand and Kramer, 2014) because NPQ actually showed the linear relationship with P700 oxidation (Figure 5). Nevertheless, we could not exclude the possible impact of O_2_ *via* FLV at the electron acceptor side of PSI. Since the contributions of FLV and photorespiration to the O_2_-dependent electron sink should be related to the amount of Rubisco in the thalli, we assume that photorespiration possibly replaces FLV to function for P700 oxidation in some specific situations.

Photorespiration is likely to already occur in cyanobacteria (Eisenhut et al., 2008) but only started to function as an alternative electron sink following adaptation of oxygenic photosynthetic organisms to terrestrial habitats (Hanawa et al., 2017), which was presumably enabled by the increase in the atmospheric O_2_ partial pressure. However, when basal land plants emerged, approximately 0.5Gayears ago, atmospheric O_2_ partial pressure was still low (*ca.* 2kPa) and CO_2_ partial pressures remained high (>1kPa; Kasting, 1987; Berner and Kothavala, 2001). Therefore, photorespiration would not have functioned at significant rates during the time when some green algae are acclimating the terrestrial conditions and evolving into the basal land plants. Such a situation possibly has been a reason why FLV plays a key role for the protection of PSI against excess light energy.

The molecular mechanism for the regulation of photosynthetic electron transport by PGR5 and PGRL1 is still under debate. PGR5 was first designated as a component essential for the antimycin-sensitive pathway of cyclic electron transport around PSI (Munekage et al., 2002). In this hypothetical model, the inability to oxidize P700 in *pgr5* mutants could be explained by dysfunctional “photosynthetic control,” due to the lack of effective ΔpH generation by cyclic electron transport (Yamori and Shikanai, 2016). One problem that should be noted is the difficulty in measuring cyclic electron transport *in vivo*. The *in vivo* activity of cyclic electron transport has been often measured using the effective quantum yield of PSI, the so-called Y(I), which is easily overestimated when abundant plastocyanin is in its oxidized form (Furutani et al., 2020a). This is the reason why high Y(I) over Y(II) values were recorded on heterologous expression of FLV in Arabidopsis and rice mutants deficient in PGR5 (Yamamoto et al., 2016; Wada et al., 2018). That is, Y(I) can be misinterpreted when P700 is kept oxidized (Furutani et al., 2020a). Another hypothetical model has been proposed for the physiological function of PGR5, based on the higher *g*_H_^+^ in the *pgr5* mutants than in wild- type plants (Kanazawa et al., 2017; Rantala et al., 2020). Since ΔpH is controlled not only by pumping H^+^ into the thylakoid lumen through photosynthetic electron transport but also by narrowing the leak of H^+^ from the lumen to the stroma (Rott et al., 2011). That is, PGR5 may be a regulatory factor for H^+^ leakage across thylakoid membranes. This model could equally explain the inability of *pgr5* mutants to oxidize P700, in the same way as the cyclic electron transport model. In *C. reinhardtii*, the amount of FNR attached to the thylakoid membrane decreased in the mutant deficient in *pgr5* (Mosebach et al., 2017). Further, it has been recently found that the *pgr5* mutant possesses the dysfunctional Cyt *b*_6_*f* complex in *C. reinhardtii*, which is assumed to be a cause of lower ΔpH in the mutant (Buchert et al., 2020). The exact molecular function of PGR5 remains to be uncovered in future work.

## Data Availability Statement

The original contributions presented in the study are included in the article/Supplementary Material, further inquiries can be directed to the corresponding author.

## Author Contributions

CM conceived the research plan. GS and HH performed all experiments. GS provided assistances to HH. SW, GH, and YM provided assistances to GS. GS, HH, and CM designed experiments and analyzed the data, and GS wrote the article with supports by GH and CM. All authors contributed to the article and approved the submitted version.

## Conflict of Interest

The authors declare that the research was conducted in the absence of any commercial or financial relationships that could be construed as a potential conflict of interest.

## Publisher’s Note

All claims expressed in this article are solely those of the authors and do not necessarily represent those of their affiliated organizations, or those of the publisher, the editors and the reviewers. Any product that may be evaluated in this article, or claim that may be made by its manufacturer, is not guaranteed or endorsed by the publisher.
