# Supplementary material for: Physiological Roles of Flavodiiron Proteins and Photorespiration in the Liverwort Marchantia polymorpha
Source: Front Plant Sci. 2021 Aug 19;12:668805. doi: 10.3389/fpls.2021.668805 (PMC8418088; doi:10.3389/fpls.2021.668805)
Supplement: Supplementary file 1 [file Presentation_1.PPTX]

## Slide 1
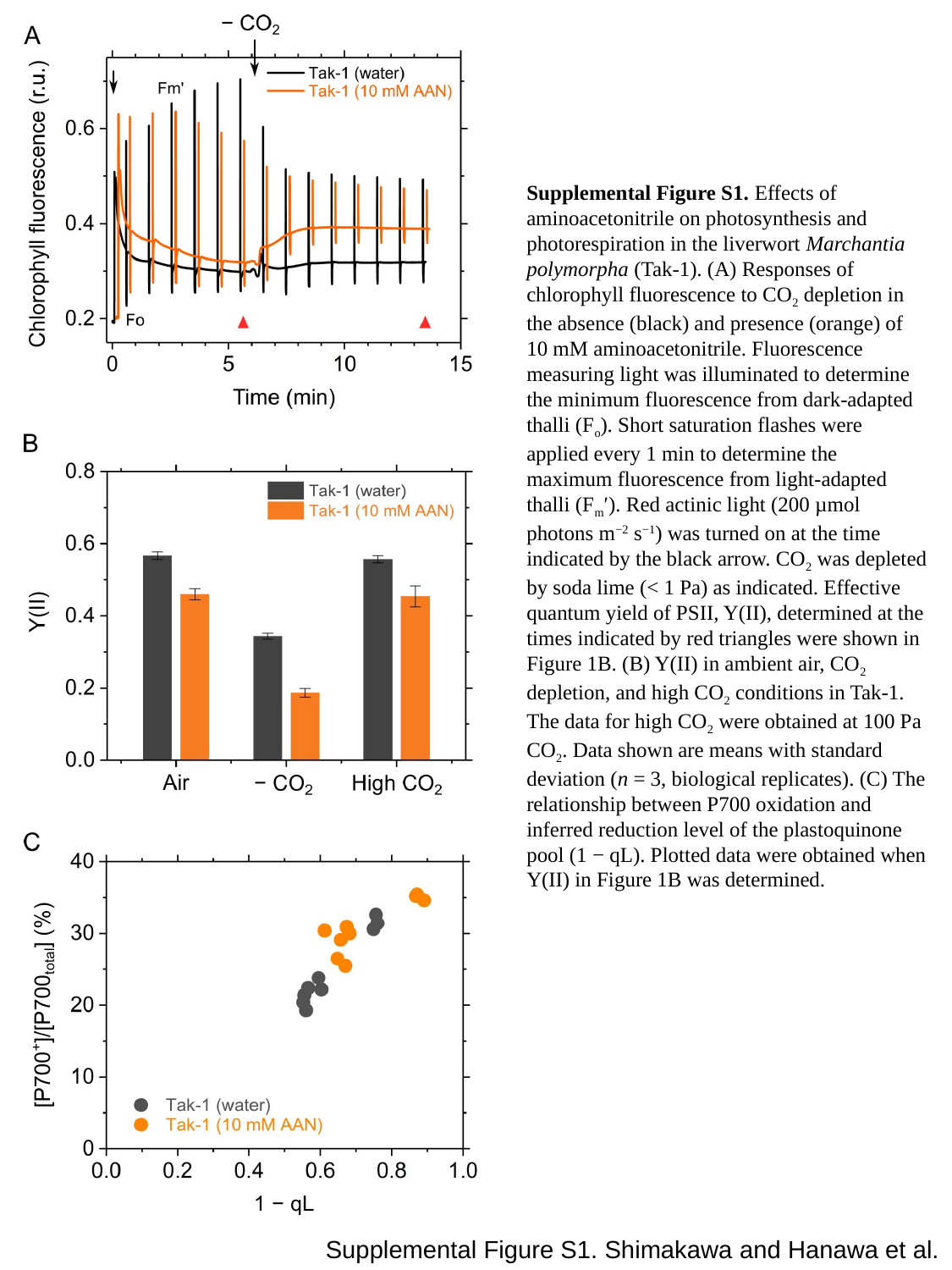

Supplemental Figure S1. Effects of aminoacetonitrile on photosynthesis and photorespiration in the liverwort Marchantia polymorpha (Tak-1). (A) Responses of chlorophyll fluorescence to CO2 depletion in the absence (black) and presence (orange) of 10 mM aminoacetonitrile. Fluorescence measuring light was illuminated to determine the minimum fluorescence from dark-adapted thalli (Fo). Short saturation flashes were applied every 1 min to determine the maximum fluorescence from light-adapted thalli (Fmʹ). Red actinic light (200 µmol photons m−2 s−1) was turned on at the time indicated by the black arrow. CO2 was depleted by soda lime (< 1 Pa) as indicated. Effective quantum yield of PSII, Y(II), determined at the times indicated by red triangles were shown in Figure 1B. (B) Y(II) in ambient air, CO2 depletion, and high CO2 conditions in Tak-1. The data for high CO2 were obtained at 100 Pa CO2. Data shown are means with standard deviation (n = 3, biological replicates). (C) The relationship between P700 oxidation and inferred reduction level of the plastoquinone pool (1 − qL). Plotted data were obtained when Y(II) in Figure 1B was determined.
Supplemental Figure S1. Shimakawa and Hanawa et al.
